# The Antiangiogenic Effect of Sanguinarine Chloride on Experimental Choroidal Neovacularization in Mice via Inhibiting Vascular Endothelial Growth Factor

**DOI:** 10.3389/fphar.2021.638215

**Published:** 2021-03-15

**Authors:** Junxiu Zhang, Ke Mao, Qing Gu, Xingwei Wu

**Affiliations:** ^1^ Shanghai Key Laboratory of Ocular Fundus Diseases, Shanghai Engineering Center for Visual Science and Photomedicine, Department of Ophthalmology, Shanghai General Hospital, Shanghai Jiao Tong University School of Medicine, Shanghai, China; ^2^ Department of Ophthalmology, Renji Hospital, Shanghai Jiao Tong University School of Medicine, Shanghai, China

**Keywords:** angiogeneisis, age-related macula degeneration (AMD), choroidal neovascluarization, sanguinarine chloride, vascular endthelial growth factor (VEGF)

## Abstract

**Background:** The purpose of this study is to investigate the antiangiogenic effect of Sanguinarine chloride (SC) on models of age-related macular degeneration (AMD) both *in vivo* and *in vitro*.

**Methods:** Choroidal neovascularization (CNV) was conducted by laser photocoagulation in C57BL6/J mice. SC (2.5 μM, 2 μl/eye) was intravitreally injected immediately after laser injury. The control group received an equal amount of PBS. 7 days after laser injury, CNV severity was evaluated using fundus fluorescein angiography, hematoxylin and eosin (H&E) staining, and choroid flat-mount staining. Vascular endothelial growth factor (VEGF) expression in the retina/choroid complex was measured by western blot analysis and ELISA kit. *In vitro*, human retinal microvascular endothelial cells (HRMECs) were used to investigate the effects of SC on cell tube formation, migration, and cytotoxicity. The expression of VEGF-induced expression of extracellular signal-regulated kinase (ERK)1/2, protein kinase B (AKT), mitogen-activated protein kinases (p38-MAPK) *in vitro* and laser induced VEGF expression *in vivo* were also analyzed.

**Results:** SC (≤2.5 μM) was safe both *in vitro* and *in vivo*. Intravitreal injection of SC restrained the formation of laser induced CNV in mice and decreased VEGF expression in the laser site of the retina/choroid complex. *In vitro*, SC inhibited VEGF-induced tube formation and endothelial cell migration by decreasing the phosphorylation of AKT, ERK1/2, and p38-MAPK in HRMECs.

**Conclusions:** SC could inhibit laser-induced CNV formation via down-regulating VEGF expression and restrain the VEGF-induced tube formation and endothelial migration. Therefore, SC could be a potential candidate for the treatment of wet AMD.

## Introduction

Age-related macular degeneration (AMD) is the leading cause of severe blindness among the elderly in developed countries (Mitchell et al., 2018). The exudative or neovascularization AMD (wet AMD), though only consist of 10–15%, is responsible for approximately 90% of the vision loss in AMD patients. Choroidal neovascularization (CNV), the hallmark of wet AMD,is represented by pathological angiogenesis originating from choriocapillaris and breaking through Bruch’s membrane as well as RPE, resulting in the subretinal space leakage and sudden vision loss (Al-Zamil et al., 2017). Though the exact mechanism of AMD is still not clear, vascular endothelial growth factor (VEGF) is well accepted to play a crucial role in the angiogenesis formation (Bhutto and Lutty, 2012; van Lookeren Campagne et al., 2014). The current standard therapy for the treatment of wet-AMD is repeated intravitreal injections of antibodies against vascular endothelial growth factor (VEGF) (Aiello et al., 1995; Ferrara et al., 2006). Though it is effective in preventing and reducing neovascularization in some patients, it also has limitations including the need for frequent intravitreal injection and inducement of macular disorders. What’s more, the current therapy is expensive and a huge burden for both patients and society (Song et al., 2020). Therefore, the replacement of the current therapy is in urgent need.

Natural herbal products or components are an important source of alternatives because of their low toxicity and low cost (Fu et al., 2018). One of the promising candidate is Sanguinarine Chloride (SC) (Mackraj et al., 2008; Basu and Kumar, 2016), a benzo phenanthridine alkaloid derived from the root of Sanguinaria Canadensis and other poppy-fumaria species, which has been proved to exert anti-tumor properties due to its anti-proliferation, antiangiogenic and anti-invasive properties in various preclinical studies (Gaziano et al., 2016; Achkar et al., 2017). Specifically, SC could inhibit angiogenesis of cancer cells via blocking VEGF-induced vessel growth, cell migration, sprouting, and survival (Galadari et al., 2017; Xu et al., 2012; Eun and Koh, 2004; Basini et al., 2007a; Basini et al., 2007c). As the fact that neovascular vessels built in ocular diseases has the similar pathology features with tumor, which are all built to compensate for the lack of oxygen (Hennig and Goepferich, 2015), we hypothesed that SC might also be a suitable antiangiogenic treatment for ocular neovascular diseases. Moreover, the effect of SC on human retinal microvascular endothelial cells (HRMECs) has not been studied yet and its potential of antiangiogenic effect in wet AMD has not been reported. Therefore, we are interested to see whether SC could inhibit the neovascularization in wet AMD models.

In this study, we used a laser induced CNV mice model, which was a classical animal model to study wet AMD. First, the safety study was performed *in vitro* using a cell counting kit (CCK8) assay and *in vivo* using HE staining assay and TUNEL assay. The fundus fluorescein angiography (FFA) assay, retina-choroid flat mount, HE staining assay were studied to evaluate the therapeutic effect of SC on CNV formation. The tube-like structure assay and scratch-wound assay in HRMECs were conducted to see whether SC could inhibit angiogenesis *in vitro*. Furthermore, the expression of VEGF protein in retina-choroid complexes and the level of AKT, ERK, p38-MAPK in HRMECs were studied to explore the possible mechanisms. To our knowledge, this is the first study to investigate the therapeutic effect of SC in ocular neovascular disease.

## Materials and Methods

### Animals

We used adult 6–8 week-old male C57BL/6J mice (purchased from the Shanghai Laboratory Animal Center of the Chinese Academy of Sciences), weighing 20–30 g for laser-induced CNV studies. Animals were housed under controlled lighting conditions (12/12 h light/dark cycle). All experiments were performed in accordance with the Association for Research in Vision and Ophthalmology Statement for the Use of Animals in Ophthalmic and Vision Research. All experiments were approved and monitored by the Institutional Animal Care and Use Committee (IACUC) at the medical academy of Shanghai Jiao Tong University.

### Fundus Fluorescein Angiography Imaging and Grading

Seven days after laser treatments, fluorescein angiography was performed following a previously described protocol (Elizabeth Rakoczy et al., 2006; Shah et al., 2015). In brief, mice were fully anesthetized, and the eyes were dilated, and a drop of lubricant gel was placed on each eye. Intraperitoneal injection of 1 ml of a 2% fluorescein sodium solution (Alcon, Fort Worth, TX, United States) was used to get a clear bright-field image. Fundus images were captured using the platform of the Micron IV fundoscopy system (Phoenix Research Laboratories, Pleasanton, CA, United States). The images were processed using Image J software and quantitatively evaluated by two blind groups of observers following four categories as described previously.

### Laser-Induced Choroidal neovascularization and Injections

Laser-induced CNV lesions were induced following a standard protocol (Xin et al., 2020). In brief, animals were first anesthetized by intraperitoneal injection of avertin (2,2,2-tribromoethanol) (0.2 ml/10 g) (Nan Jing AIBI Bio-Technology Co., Ltd, Nanjing, China). Their pupils were dilated with 0.5% tropicamide (Santen Pharmaceutical, Osaka, Japan). A drop of levofloxacin gel (Alcon) was placed on the eye to avoid any eye dehydration and infection. Four laser photocoagulations (532 nm, 350 mW, 100 ms) were implemented in the fundus of each eye surrounding the optic nerve and focused on the Bruch’s membrane using a Micron IV retinal image-guided laser system (Phoenix Research Laboratories). The morphologic endpoint of the laser injury was the appearance of an air bubble, which is a sign of BM disruption. Intravitreal injections were performed following earlier protocols (Mitra et al., 2017). In brief, immediately after laser injury, a 30-gauge needle was used to make a precise hole on the sclera of each eye. A 35-gauge needle attached to a 10 μl nanofilm syringe (World Precision Instruments, Sarasota, FL, United States) was then gently inserted through the puncture hole into the vitreous cavity delivering 2 μl of SC (Selleckchem, Cat. No. S5452) at the concentration of 2.5 μM or PBS solution. The injection process was visualized using an operating microscope (Carl Zeiss Surgical, Incorporated, Thornwood, NY, United States).

### Choroidal Flat Mounting and Quantification of Choroidal Neovascularization

We measured CNV volume as previously described (Mao et al., 2017; Wei et al., 2018; LeBlanc et al., 2019). In brief, 7 days after laser photocoagulation, we sacrificed the mice and enucleated the eyeballs. After fixing the eyeballs in 4% paraformaldehyde (Electron Microscopy Sciences [EMS], Hatfield, PA) for 1 h, we removed the corneas, the lenses, the vitreous, and the neurosensory retinas. The choroid/RPE/scleral tissue was isolated with four radial incisions and washed three times in PBS. Then we incubated the processed choroid/RPE/scleral complexes with blocking buffer (PBS containing 1% BSA and 0.5% Triton X-100) for 4 h at room temperature. After washing three times in PBS, the RPE complexes (RPE/choroid/sclera) were stained with 10 μg/ml Alexa Fluor^®^488 conjugated isolectin GS-IB4 (Invitrogen, Cat. No. 121411) in a volume of 0.5 ml/retina overnight at 4°C and flat-mounted with antifade mounting medium (VECTASHIELD; Vector Laboratories, CA) to preserve the fluorescence. We observed CNV with a Zeiss LSM 710 confocal laser scanning microscope (Carl Zeiss) at a magnification of 100×. An average of 3–4 CNV areas per eye was analyzed. Each CNV spot was considered an independent data point. For each group, at least 25 spots were analyzed from eight mice. All quantifications were conducted under blinded conditions.

### Histopathological Examination and the Terminal Deoxynucleotidyl-Transferase-Mediated dUTP Nick-End Labeling Assay

Hematoxylin and eosin (H&E) staining was performed according to a previously described procedure (Wei et al., 2018; Xin et al., 2020). Mice were killed, and the eyes were enucleated 3°days (for safety test) or 7°days (for CNV volume analysis) after laser injury and fixed in 4% paraformaldehyde at 4^°^C for 24°h, conventionally dehydrated, and embedded in paraffin wax. The fixed tissues were serially sectioned at 3 μm, stained with HE. The tissues were observed and photographed under a light microscope. The TUNEL assay (*In situ* cell death detection kit-POD; Roche Diagnostics GmbH, Mannheim, Germany) was performed according to the manufacturer’s instructions 3 days after laser injury and was observed under a fluorescence microscope at 200× magnification.

### Cell Culture

The HRMECs were purchased from the Cell Bank of the Chinese Academy of Sciences and cultured in endothelial cell medium (ECM) containing 5% fetal bovine serum (FBS, Sigma, and Cat. No. F2442), 1%penicillin/streptomycin and 2% endothelial cell growth supplement (ECGS) at 37°C in a humidified incubator with 5% CO_2_. HRMECs cells were cultured in serum-free medium (SFM) for 24 h before treatments.

### Cell Viability Assay

Cell viability was analyzed using a Cell Counting Kit (CCK)-8 (Dojindo Molecular Technologies). HRMECs were seeded in 96-well plates at a density of 10,000 cells/well and incubated at 37°C in a 5% CO_2_ environment for 24 h. The culture media from each well was then removed and replaced with 100 μl of a serum-free medium for another 24 h to starve the cells. Then, serum-free medium containing different concentrations (1, 2.5, 7.5, 10, and 20 μM) of SC was added into each well, and the plates were continuously incubated for different times (24 , 48 h). A 10 μl volume of CCK8 solution was gently added per well and incubated at 37°C for 4 h. The absorbance was measured at 492 nm using a microplate reader. Untreated cells served as negative controls, and only media without cells served as background measurements. The cell viability was calculated following the manufacturer’s protocol. All experiments were performed in triplicate.

### Tube Formation Assay

HRMECs were used for the tube formation assay, as described previously (Mao et al., 2017; Mitra et al., 2017). In brief, Corning Matrigel matrix (Matrigel, Becton Dickinson, NJ, United States) was placed in a 96-well plate at 37°C for 45 min to allow the basement membrane to form a gel. Then 5 × 10^4^ HRMECs were transferred to each well with or without treatment with varying concentrations of SC and VEGF (10 ng/ml). 2-Methoxyestradiol (2-Me, a metabolite of estradiol-17beta) (Pal et al., 2019) at the concentration of 10 nM was used as a positive control. The plate was incubated for 6 h at 37°C with 5% CO_2_. Capillary-like tube structures formed by HRMECs on the Matrigel were photographed with a Leica CTR6000 fluorescence microscope (Wetzlar, Germany). Tube formation was quantified by counting the average number of branches per field. The experiments were repeated at least three times.

### The Scratch Wound-Healing Assay

HRMECs were seeded into 24-well tissue culture plates and cultured for 24 h until approximately 70–80% confluence. After replacing the medium with serum-free ECM for 24 h to starve the cells, we used a sterile 1,000 μl pipette tip to make a straight line on the monolayer across the center of the well in a single direction. Then Similarly, a second straight line was scratched perpendicular to the first line to create a cross-shaped cellular gap in each well. Each well was washed twice with 1 ml of cold PBS to remove the debris and any detached cells. before changed the medium with serum-free medium (control group), 2.5 μM SC or 10 nM 2-Me (positive control) for another 24 h. All cells were incubated at 37°C in a humidified atmosphere containing 5% CO_2_ for 24 h before capturing digital images of the cell gaps using a Leica CTR6000 fluorescence microscope (Wetzlar, Germany).

### ELISA Assay

The concentration of VEGF in RPE-choroid complexes was performed 3°days after laser burn with a mouse ELISA kit (R&D Systems, United States) according to the manufacturer’s instruction.

### Western Blot Analysis

Western blotting was performed following the previous protocol (Mao et al., 2017). HRMECs or isolated retina/choroid complexes were lysed in modified RIPA buffer (Thermo, Rockford, IL, United States) supplemented with protease inhibitor cocktail (Thermo, Rockford, IL, United States) on ice. Proteins obtained from the cell lysates were separated on 10% sodium dodecyl sulfate-polyacrylamide gel electrophoresis and transferred to nitrocellulose (NC) membranes (Millipore, Billerica, MA). Membranes were blocked in Tris-buffer saline Tween 20 buffer containing 5% nonfat milk for 1 h at 37°C, and immunoblotted with the following primary antibodies: rabbit anti-VEGF-A (ab46154, Abcam; 1:1,000), anti-phospho-extracellular signal-regulated kinases (ERK1/2) (Cell Signaling, Danvers, MA, United States), anti-ERK1/2 (Cell Signaling), anti–phospho-protein kinase B (AKT) (Cell Signaling), anti-AKT (Cell Signaling), anti–Phospho-p38 MAPK (Cell Signaling), anti-p38 MAPK (Cell Signaling), and *β*-actin(Cell Signaling) at 4°C overnight. Membranes were then washed with TBST and incubated with the corresponding secondary antibodies at 37°C for 2 h. The membranes were washed with TBST three times to remove the unlinked secondary antibodies and the chemiluminescence visualized following the manufacturer’s instructions using an enhanced chemiluminescence (ECL) reagent (Pierce, Rockford, IL, United States) with a ChemiDoc MP Imager (BIO-RAD). Each indicated band was quantified and normalized to the corresponding loading control using Image J software.

### Statistical Analysis

The experimental data were presented as the mean ± SEM in figures. GraphPad Prism 8.0 software (La Jolla, CA, United States) was used for the figures and data analysis and Photoshop CS5 software for figures organization. T-tests were used for the comparisons between two groups, while multiple group comparisons were analyzed using one-way and two-way ANOVA as appropriate. A *p* value of less than 0.05 was considered to be significant.

## Results

### Low Concentration of SC Showed No toxicity Both *In Vitro* and *In Vivo*

**FIGURE 1:**
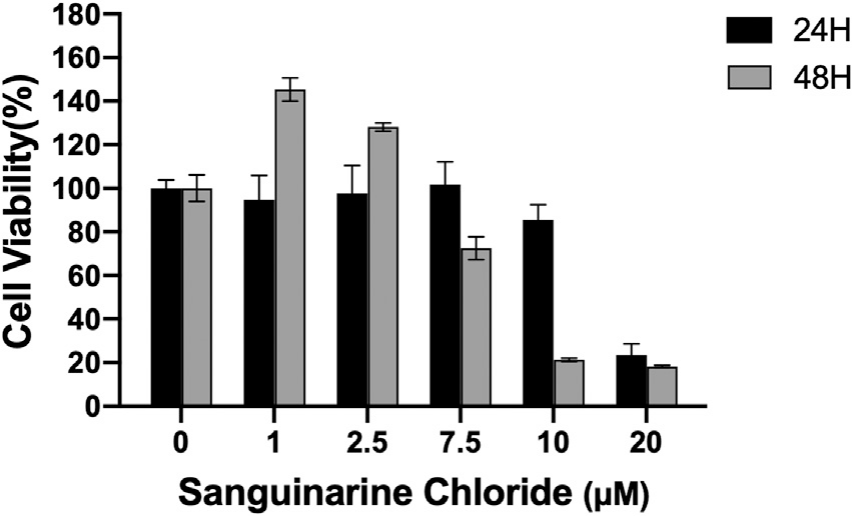
Quantitative assessment of *in vitro* cell viability in human retinal microvascular endothelial cells (HRMECs). HRMECs were incubated with different concentrations of Sanguinarine Chloride (SC) (0, 1, 2.5, 7.5, 10, 20 μM) for 24 and 48 h; cell viability was measured using the CCK8 assay.

CCK8 assay was used to evaluate the cytotoxicity of SC toward HRMECs. As shown in Figure 1, low concentrations of SC (≤2.5 μM) produced no inhibition on the growth of HRMECs from day 1 to day 2. However, SC (>2.5 μM) began to affect the survival of HRMECs cells in a concentration- and time-dependent manner. Therefore, we chose lower concentrations of SC (≤2.5 μM) for further research.

### Inhibition of Human Retinal Microvascular Endothelial Cells Tube Formation and Migration by Sanguinarine Chloride

**FIGURE 2:**
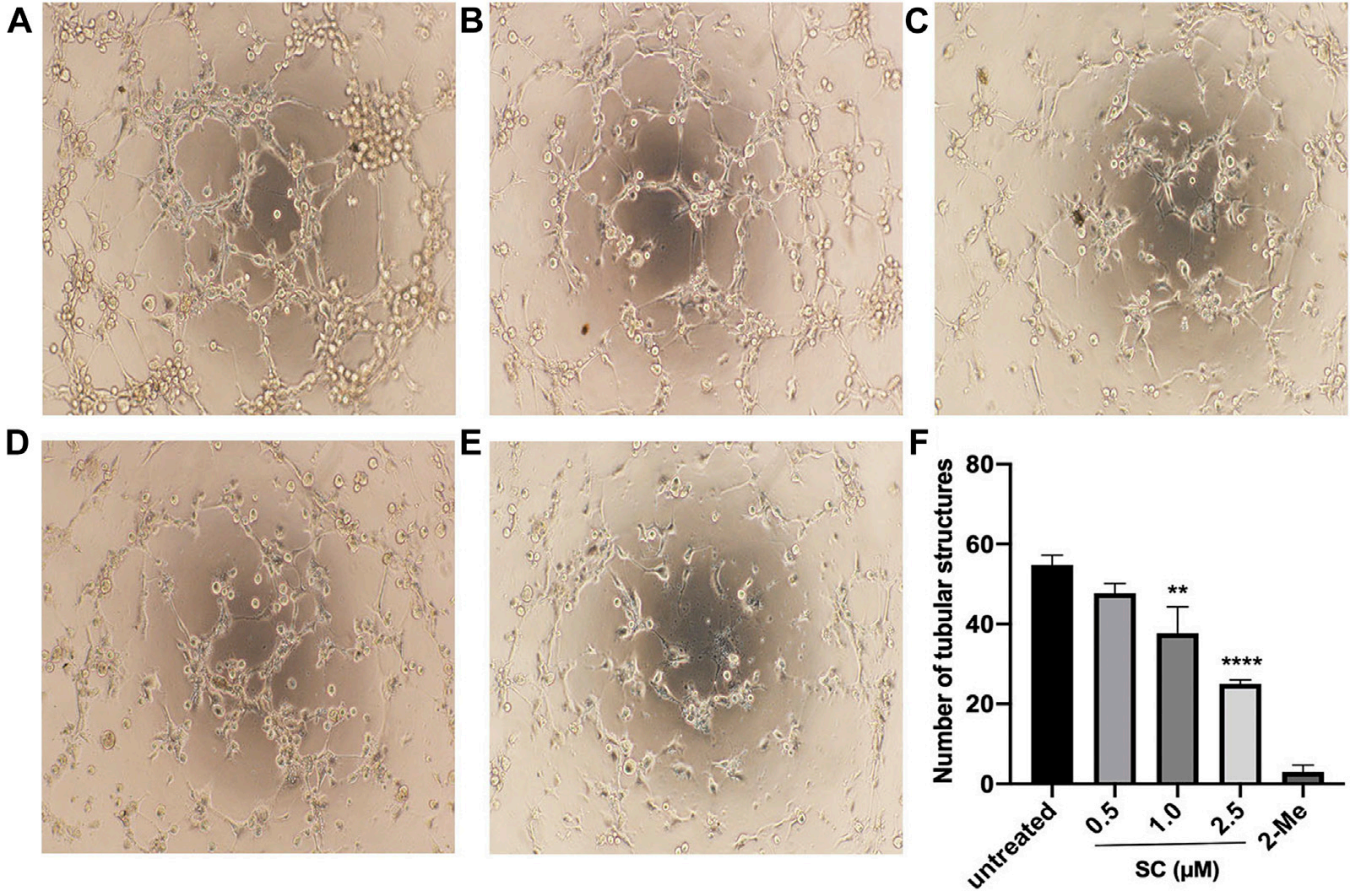
Anti-angiogenic effect of SC in HRMECs. Tube-like structures were captured after HRMECs incubated with vascular endothelial growth factor (VEGF) 10 ng/ml supplemented with **(A)** no SC (untreated group), **(B)** 0.5 μM SC, **(C)**1.0 μM SC, **(D)** 2.5 μM SC or **(E)**10 nm 2-Methoxyestradiol (2-Me) (positive control) for 6 h. **(F)** Quantitative analyses of the number of tubular structures. A **p-value <0.01 was considered significant compared to untreated group. ****p-value <0.0001 vs. untreated group.

To investigate the effect of SC on the process of angiogenesis, we performed a VEGF-induced capillary tube formation assay using HRMECs. As can be seen in Figure 2, VEGF treatment led to robust tube formation. 2-Me, an angiogenic inhibitor of HRMECs proliferation and angiogenesis, was used as a positive control in this test and showed a strong inhibitory effect on tube formation. However, SC treatment significantly decreased the number of tubular-like structures in a concentration-dependent manner. Compared to the VEGF-treatment group, the number of capillary-like structures was reduced by 9.76%, 32.02% and 64.45% on the treatment on 0.5, 1, and 2.5 μM SC group, respectively.

**FIGURE 3:**
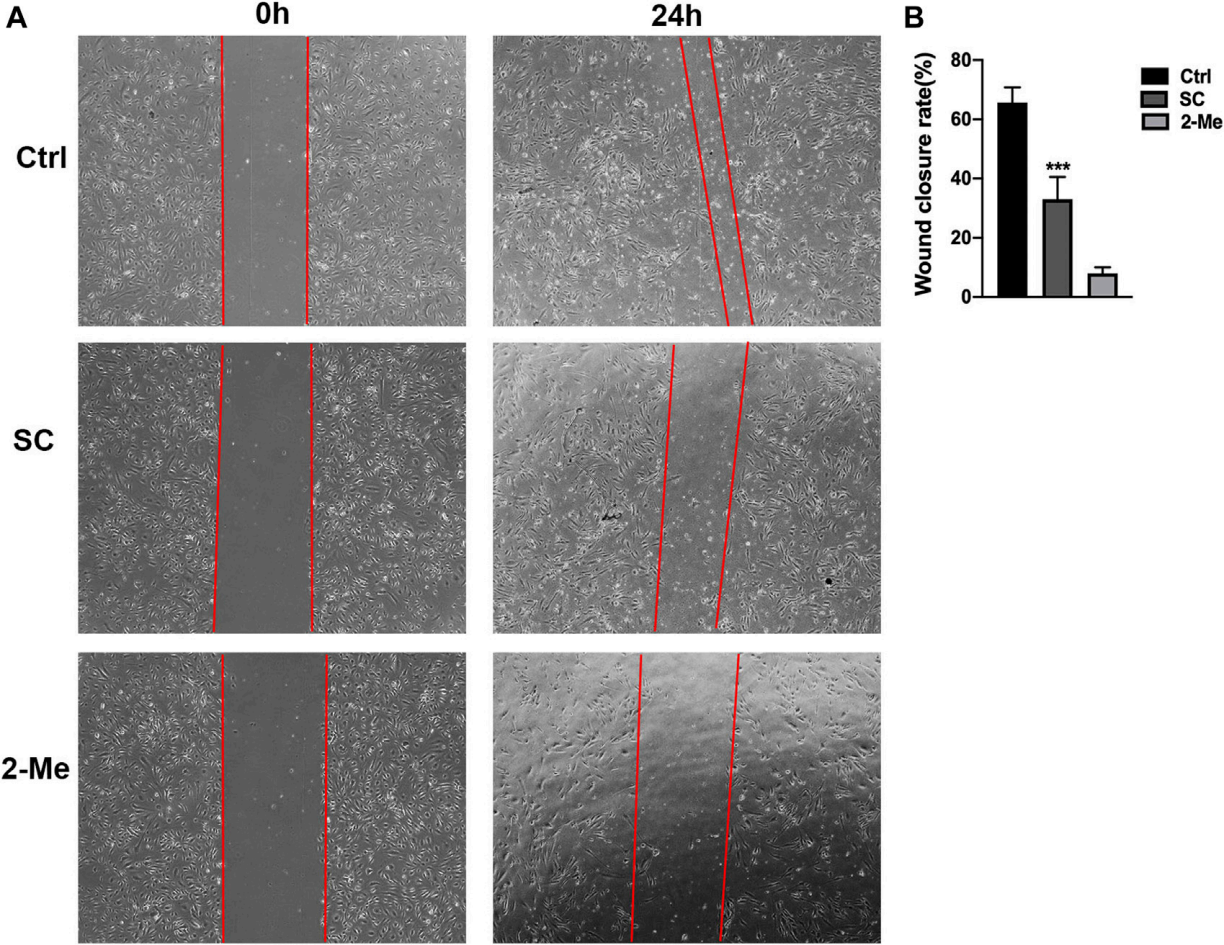
SC significantly delayed wound healing of HRMECs. The scratch-wound assay was performed on monolayer HRMECs cultures using a 1 ml pipet tip. **(A)** Representative photographs were captured at 0 and 24 h after wounding. The migration distances of HRMECs at the edge of the scratch wound were recorded under microscope. **(B)** Quantification analysis of HRMECs migration rates. The migration distance of SC treated group was significantly less than the untreated (Ctrl) group, ***p < 0.001 vs. control group. The results represent the mean ± SEM (*n =* 3) of one-way ANOVA followed by Tukey’s *posthoc* multiple comparison tests.

As the migration of abnormal endothelial cells plays a crucial role in angiogenic process, we further elevated the effect of SC on HRMECs migration using the scratch-wound assay. As shown in Figure 3, SC treatment reduced 49.75% of wound closure rate compared to the untreated group, which is consistent with the tube formation assay. Our results are in line with the earlier study where SC exhibited a significantly anti-angiogenic effect in various kinds of tumors (Gaziano et al., 2016; Galadari et al., 2017).

### Intravitreal Injection of Sanguinarine Chloride Attenuates Choroidal Neovascularization Development

**FIGURE 4:**
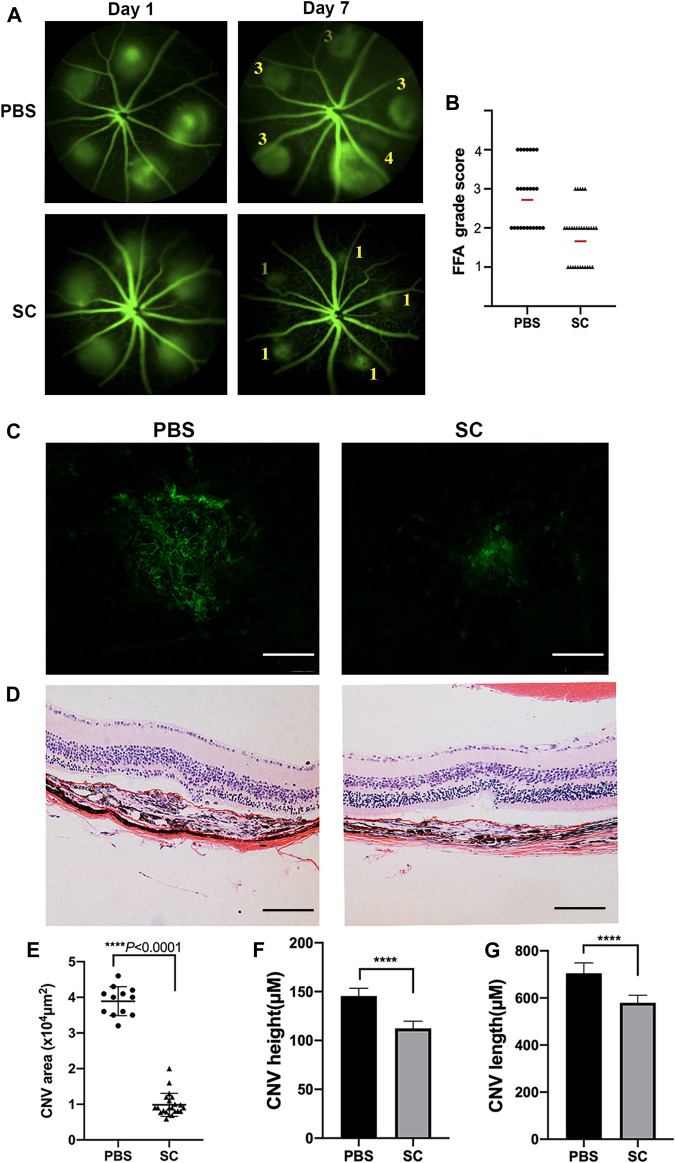
The intravitreal injection of SC significantly decreased Choroidal neovascularization (CNV) size in mice. **(A)** Fluorescein angiograms of the eye fundus captured on Day 1 and Day 7 after laser damage with the intravitreal injection of SC (2.5 μM, 2 μl/eye) or the equal amount of PBS. The numbers in the photograph represent the grade of vascular leakage. **(B)** Quantitative analysis of fundus fluorescein angiography (FFA) grade scores of each group (black dots or triangles); the red line represents average FFA grade scores. Data are presented as the means ± SEM (PBS: *n =* 8, 25 laser spots; SC: *n =* 9, 30 laser spots). **(C)** Representative images from laser induced CNV Retinal pigment epithelial cells (RPE)/choroid/scleral flat mounts 7 days after intravitreal injection of PBS (2 μl) and SC (2 μl of 2.5 μM). **(D)** Representative Hematoxylin and eosin (HE) staining microscopic images of the CNV of choroidal neovascular lesion cross-sections 7 days after photocoagulation. The scale bar represents 100 μM. **(E)** Quantitative measurements of laser induced CNV areas (×10^4^ μm^2^, PBS, 12 different laser spots; SC, 22 different laser spots), **(F)** CNV height, and **(G)** CNV length. Results are presented as the means ± SEM and analyzed with two-tailed unpaired Student’s t-tests, ****p < 0.0001.

To evaluate the inhibitory effect of SC on CNV formation in mice, we first examined the fundus fluorescein angiography (FFA) immediately after laser surgery to confirm the success of the laser burns. Injuries accompanied by choroidal hemorrhage during photocoagulation were excluded. Only the successful laser injuries were included. Then we performed a single, intravitreal injection of SC (2.5 μM, 2 μl/eye) or the equal amount of PBS immediately after photocoagulation in mice. The proliferation of new blood vessels from laser-induced injuries was considered to reach maximum damage at 7 days after laser photocoagulation (Mitra et al., 2017), so we captured the FFA images (Figure 4A) and quantitatively compared the FFA grade score of CNV lesions in each group (Figure 4B). The average FFA grade score of SC treatment group was significantly smaller than that of PBS-treated mice on Day 7 post laser injury. These data demonstrated that intravitreal injection of SC inhibited laser induced CNV formation in mice.

To reconfirm the CNV volume of different groups, we performed RPE/choroidal flat mounts with Alexa Fluor-488-conjugated *Griffonia simplicifolia* isolectin-IB4 (GS-IB4, Thermo Fisher Scientific, Cat. No. 121411) staining to identify neovascularization and measure CNV area after laser injury (Figure 4C). Twelve (PBS-treated mice) and 22 (SC-treated mice) laser spots out of the respective 50 laser spots were evaluated for endothelial cell marker staining (Figure 4E). The images of each antibody-stained CNV lesion were quantitatively analyzed using Zeiss AxioVision software, which outlined the fluorescent and antibody-stained blood vessels as determined by earlier literature (Mitra et al., 2017). The extent of the isolectin B4-labeled area was much smaller in the SC-treated group than in the PBS-treated group (*p* < 0.05).

In the HE stainig assay of morphologic cross sections (Figure 4D), CNV in the PBS group was significantly longer and higher than those in the SC group height: PBS: 142.69 ± 15.32 (μm) vs. SC: 109.12 ± 15.48 (μm), *p* < 0.05 (Figure 4F); (length: PBS: 703 ± 43.1 (μm) vs. SC: 589.59 ± 34.8 (μm), *p* < 0.05 (Figure 4G), *n =* 20 laser spots/group.

### Intravitreal Injection of Sanguinarine Chloride Inhibited Vascular Endthelial Growth Factor Expression in the Retina/Choroid Complex

**FIGURE 5:**
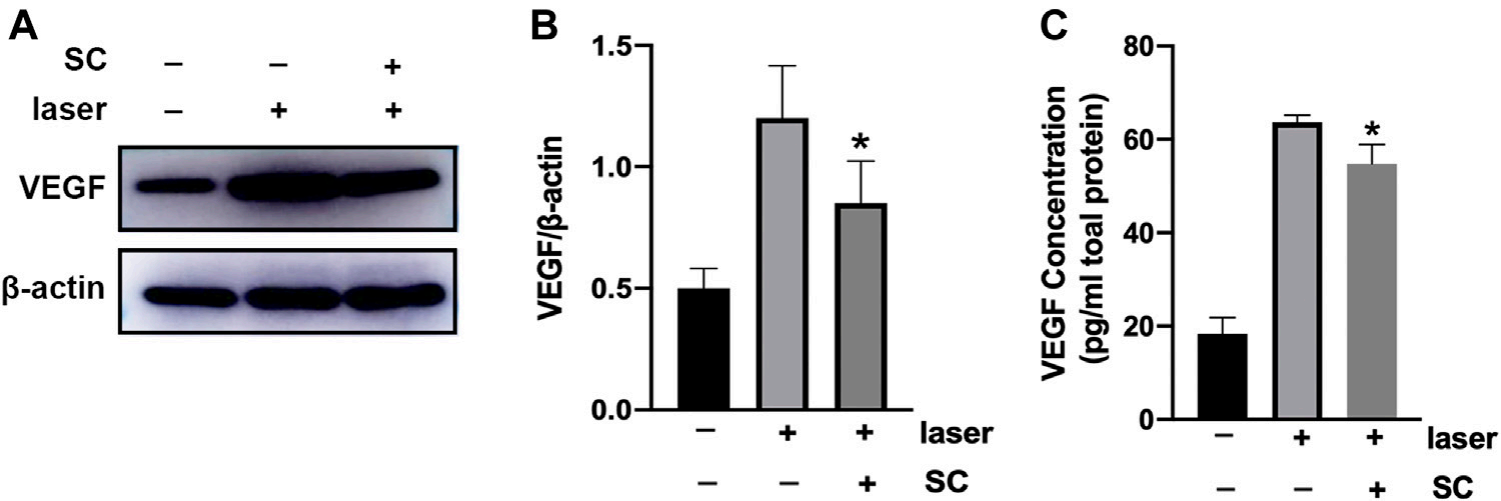
SC suppressed the expression of VEGF in retina/choroid complex. The **(A)** Immunoblotting of VEGF expression in the retina/choroid complexes was performed 7 days after laser injury with or without the treatment of SC (2.5 μM, 2 μl/eye). The group without laser injury and SC was set as control group. (*n =* 18 eyes/group) **(B)** Relative expressions of VEGF to *β*-actin. **(C)** VEGF protein level normalized to total protein concentration measured by ELISA assay in the retina-choroid complex at the same time point with the immunoblotting analysis. Pretreatment of SC significantly decrease the VEGF expression in the laser site. Data were presented as the mean ± SEM and analyzed using a one-way ANOVA followed by Bonferroni’s *posthoc* multiple comparison tests. *p < 0.05 vs. the laser group.

Seven days after intravitreal injection of SC (2.5 μM, 2 μl/eye), the amount of VEGF protein in the retina/choroid complex was evaluated using Western Blotting (Figure 5A). It was revealed that laser injury increased VEGF protein expression in the retina/choroid complex. However, pretreatment of SC could significantly reduce the expression of VEGF in the retina/choroid complex of laser induced CNV mice (*p* < 0.05) (Figure 5B).

We next evaluated the VEGF protein in the retina/choroid complex obtained from the eye up using a mouse ELISA kit. As shown in Figure 5C, the amount of VEGF in SC-treated group was significantly lower than that in PBS group (*p* < 0.05).

### Intravitreal Injection of SC Did Not Induce Toxicity to Ocular Tissue

**FIGURE 6:**
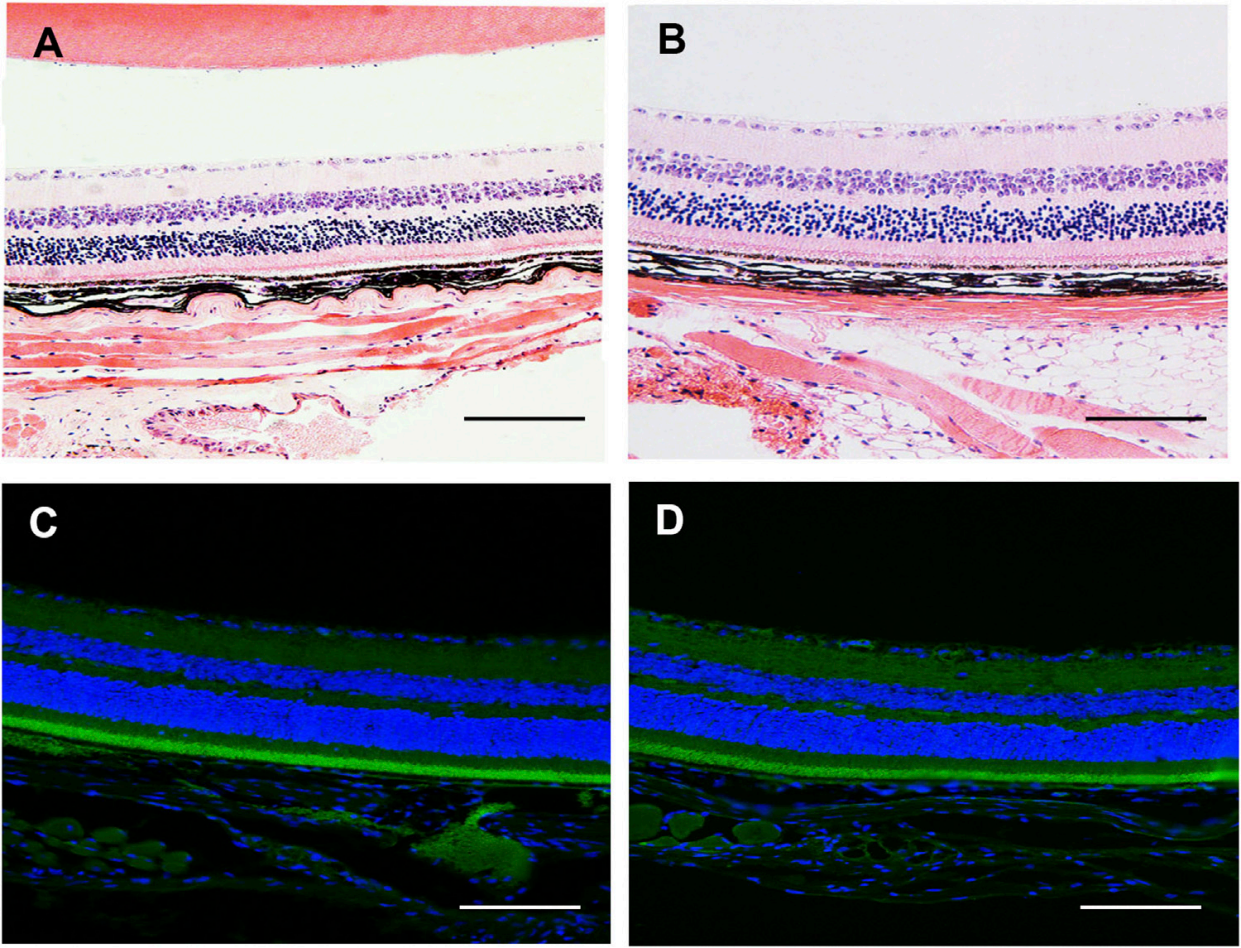
The *in vivo* safety study of SC in mice. **(A,B)** Photomicrograph of HE stained section of retina/choroid/scleral complexes 3 days after intravitreal injection of SC (2.5 μM, 2 μl/eye). No retina abnormalities or inflammatory cells were observed in each group. **(C,D)** Representative the terminal deoxynucleotidyl-transferase-mediated dUTP nick-end labeling (TUNEL) assay was performed in the retina/choroid/scleral complexes of PBS and SC group at 3 days post intravitreal injection. The nuclei were stained with 2-(4-Amidinophenyl)-6-indolecarbamidine dihydrochloride (DAPI). No apoptotic cells were detected in each group. Scale bar = 100 μM.

Before performing *in vivo* experiment, we first examined the safety of intravitreal injection of SC (2.5 μM, 2 μl/eye) in CNV eyes at 3 days after photocoagulation using HE staining and TUNEL assay. As shown in Figures 6A,B, no retina abnormalities and inflammatory cells were observed in SC-treated eye and PBS-treated eyes. The TUNEL assay (Figures 6C,D) revealed that there was no difference in TUNEL positive cells between SC group and PBS group.

### Sanguinarine Chloride Suppressed Vascular Endthelial Growth Factor-Induced Phosphorylation of Anti–Phospho-Protein Kinase B, p38-MAPK and Extracellular Signal-Regulated Kinase 1/2

**FIGURE 7:**
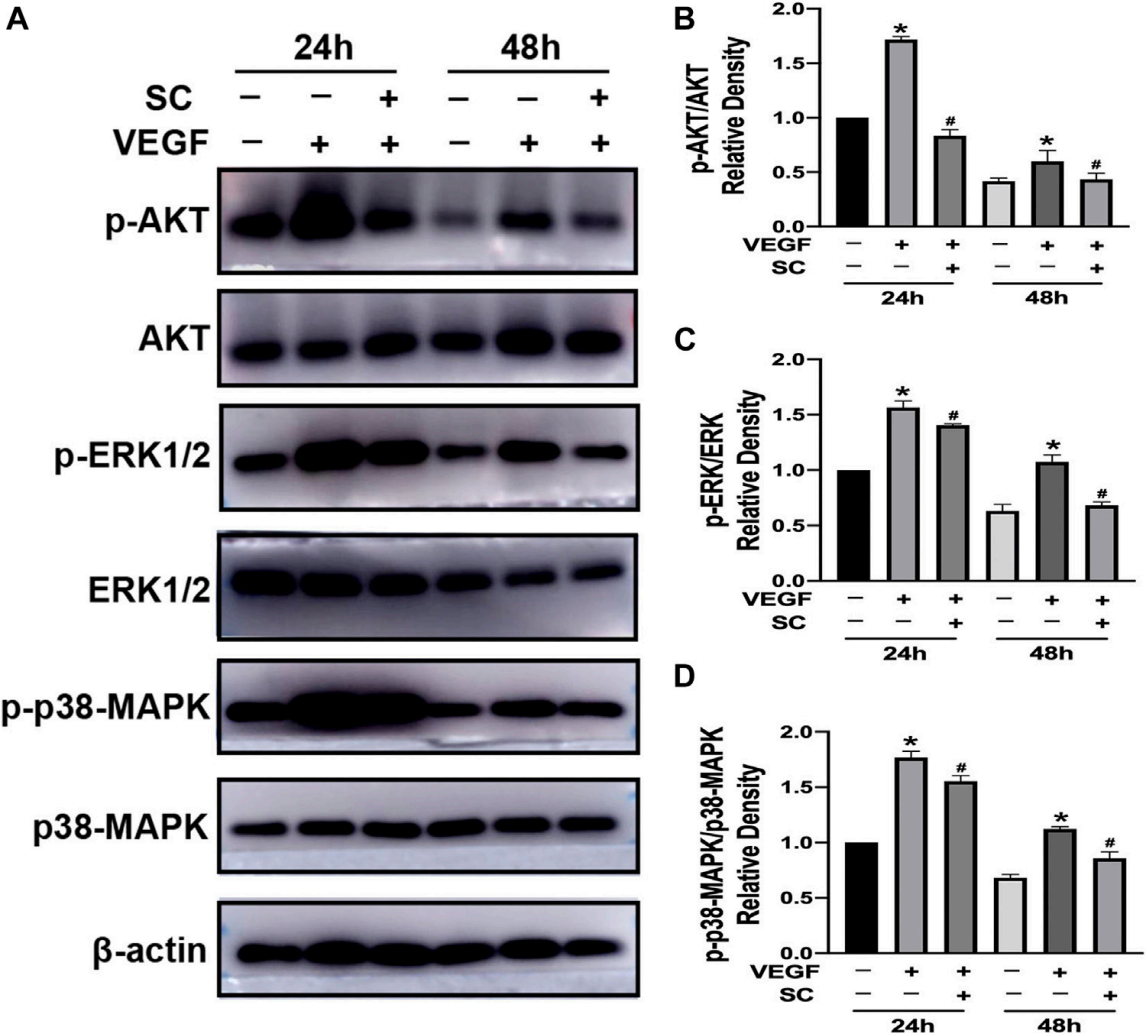
SC inhibited VEGF-induced phosphorylation of ERK1/2, Akt, and p38-MAPK in HRMECs.**(A)** The cells were first incubated with or without SC (2.5 μM) for 8 h and then treated with VEGF(10 ng/ml) for 24 and 48 h. The phosphorylation and total ERK1/2, AKT, and p-38 MAPK were analysed by Western blot. **(B–D)** The relative density ratio of p-AKT to AKT, p-ERK to ERK and p-p38-MAPK to p38-MAPK. *p < 0.05, compared to the untreated group at the corresponding time point. #*p* < 0.05 compared to the VEGF-treated group.

AKT, p38-MAPK, and ERK1/2 are crucial factors triggered by VEGF to induce the angiogenesis process, including survival, migration and proliferation of endothelial cells (Aiello et al., 1995; Roh et al., 2016; Cheng et al., 2017; Zhang and Huang, 2019). Thus we investigated the effect of SC on VEGF-induced phosphorylation of AKT (p-AKT), p38-MAPK (p-p38-MAPK), and ERK1/2 (p-ERK1/2) using HRMECs (Figure 7A). As shown in Figures 7B–D, VEGF (10 ng/ml, 24, and 48 h exposure) significantly increase the phosphorylation of AKT, p38-MAPK, and ERK1/2. However, the pretreatment of SC (2.5 μM) significantly reduced the VEGF-mediated phosphorylation of AKT, ERK1/2 (p-ERK1/2) and p38-MAPK at all time points.

## Discussion

In the present study, we revealed that intravitreal injection of SC could inhibit the development of laser induced CNV formation in mice models. SC exerted an inhibitory effect on the experimental CNV via downregulating the expression of VEGF *in vivo*. *In vitro,* SC showed an inhibitory effect on VEGF-induced endothelial tube formation and migration assay via suppressing VEGF induced AKT, ERK, p-38 MAPK signaling pathway in HRMECs. Without any toxicity observed *in vivo* at the indicated dose, SC might be a suitable antiangiogenic therapy in treating wet AMD.

It is well-known that angiogenesis plays a key role in the pathological development of neovascular ocular diseases (Bhutto and Lutty, 2012; Hellström et al., 2013; Lechner et al., 2017). Currently, the gold treatment of these neovascular eye diseases is the FDA-approved targeting VEGF approach (Mitchell et al., 2018). Though it is highly beneficial, the frequent intravitreal administration is associated with severe side-effects such as infection, bleeding, endophthalmitis, inflammation, and retinal detachment. Besides, the huge economic burden for both patients and society should not be ignored (Ferrara et al., 2006; Song et al., 2020). Our current study offered an optional, effective, and inexpensive alternative treatment for the treatment of wet AMD.

Sanguinarine chloride (SC), a potent antiangiogenic natural product, has been demonstrated to be effective due to its antiangiogenic, antiproliferation, and anti-invasive properties in various cancers including skin, breast, pancreatic, and lung malignancies (Xu et al., 2013; Gaziano et al., 2016; Galadari et al., 2017). However, its effects on neovascular ocular diseases are still unknown. Our study revealed that SC under 2.5 μM did not induce any toxicity both *in vivo* and *in vitro*. Intravitreal injection of SC (2.5 μM, 2 μl) could reduce laser induced CNV size and volume. Besides, SC downregulated VEGF expression in the retina-choroid complexes in mice, which indicated its anti-VEGF efficacy *in vivo*, in line with the previous studies (Basini et al., 2007).

SC has been reported to suppress VEGF-induced Akt phosphorylation in many conditions (Basini et al., 2007b; Galadari et al., 2017), however, the effect of SC on ERK was controversial (Li et al., 2013; Cheung et al., 2017; Zhang et al., 2017). In this study we found that SC significantly reduced the VEGF induced phosphorylation of AKT (Ser473), ERK1/2 and p38 MAPK in HRMECs, which play significant roles during angiogenesis. AKT signaling pathway is crucial in promoting endothelial cell survival while ERK1/2 and p38 (Tyr182) are vital in proliferation and migration respectively (Basini et al., 2007c; Cheng et al., 2017). This result was in line with several previous studies that SC demonstrated antiangiogenic and anti-invasive effect (Cheng et al., 2017; Zhang and Huang, 2019). However, our result was different from the previous study (Eun and Koh, 2004), which reported that Sanguinarine suppressed VEGF-induced Akt phosphorylation but not ERK 1/2 or PLCr1 phosphorylation. The difference might be the result of different concentrations of SC and different cells we chose in our studies. As reported previously, the dose of SC was closely related to its effect in different conditions (Galadari et al., 2017). The exact mechanism of SC in HRMECs and CNV models requires more research in the future.

Although our study revealed the antianigogenic effect of SC in CNV models and indicated its potential implication in wet AMD, there are some limitations must be stressed out. First, according to the *in vitro* result, we injected a relatively low concentration of SC (2.5 μM) to avoid the potential toxicity *in vivo*. However, higher concentrations are required to study the effect of SC on neovascular vessels formation. Moreover, the other possible molecular mechanisms of SC on ocular diseases, such as its effect on inflammation and oxidative stress, need to be elucidated. Furthermore, here we only studied the effect of SC in laser induced CNV mice model, however the other neovascular ocular diseases such as diabetic retinopathy and retinopathy of prematurity (Cheung et al., 2010; Hellström et al., 2013) might also be effective, which need further study. Last but not least, our study is lack of the comparison with the current standard anti-VEGF therapy, such as bevacizumab, aflibercept, ranibizumab and pegaptanib in the *in vivo* and *in vitro* experiments, which also requires further research.

In summary, our study demonstrated that SC inhibited laser-induced CNV formation and down regulated VEGF expression in experimental mice model and blocked VEGF-induced the phosphorylation of Akt, ERK1/2 and p38 MAPK in HRMECs. This study offered a potential treatment for wet AMD.

## Data Availability

The original contributions presented in the study are included in the article, further inquiries can be directed to the corresponding author.
